# Supplementary material for: Cathepsin S Evokes PAR2-Dependent Pain in Oral Squamous Cell Carcinoma Patients and Preclinical Mouse Models
Source: Cancers (Basel). 2021 Sep 19;13(18):4697. doi: 10.3390/cancers13184697 (PMC8466361; doi:10.3390/cancers13184697)
Supplement: Supplementary file 1 [file cancers-13-04697-s001.zip › cancers-1381532-supplementary.pdf]

Supplementary Materials: Cathepsin S Evokes PAR2-Dependent Pain in Oral Squamous Cell Carcinoma Patients and Preclinical Mouse Models

Nguyen Huu Tu, Kenji Inoue, Elyssa Chen, Bethany M. Anderson, Caroline M. Sawicki, Nicole N. Scheff, Hung D. Tran, Dong H. Kim, Robel G. Alemu, Lei Yang, John C. Dolan, Cheng Z. Liu, Malvin N. Janal, Rocco Latorre, Dane D. Jensen, Nigel W. Bunnett, Laura E. Edgington-Mitchell and Brian L. Schmidt

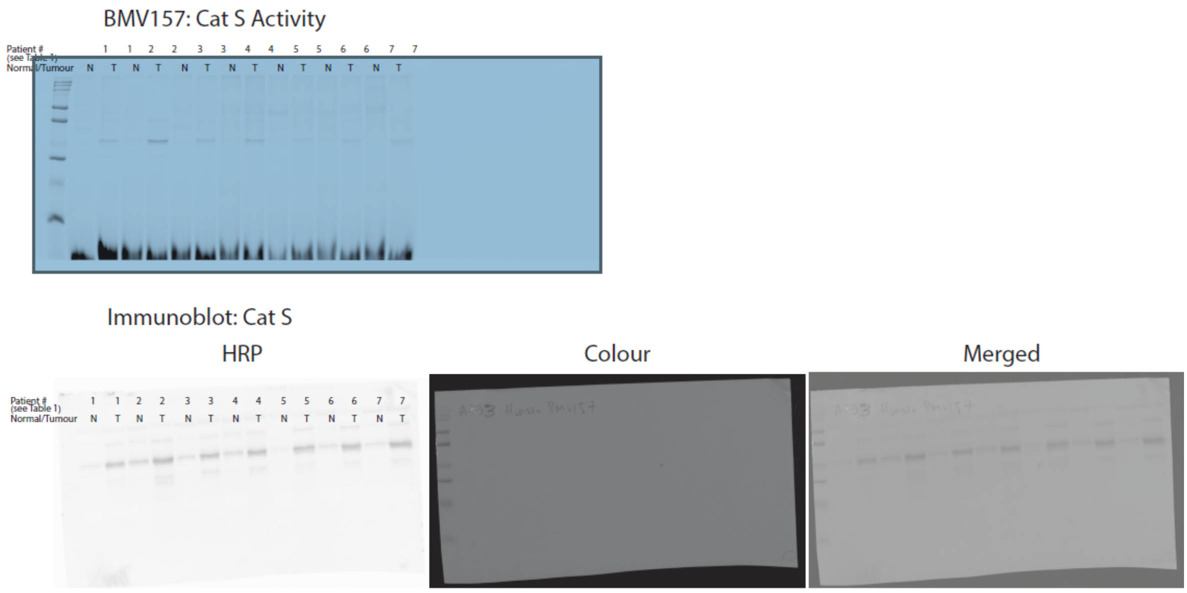

Figure S1. Uncropped western blot figures of Figure 1a.

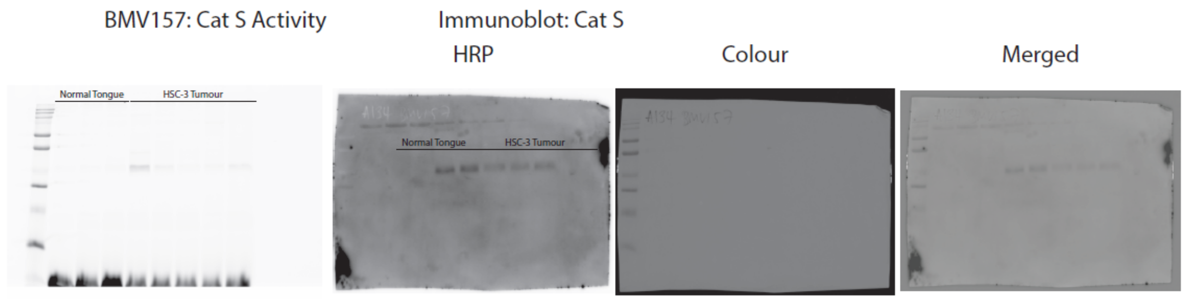

Figure S2. Uncropped western blot figures of Figure 5a.

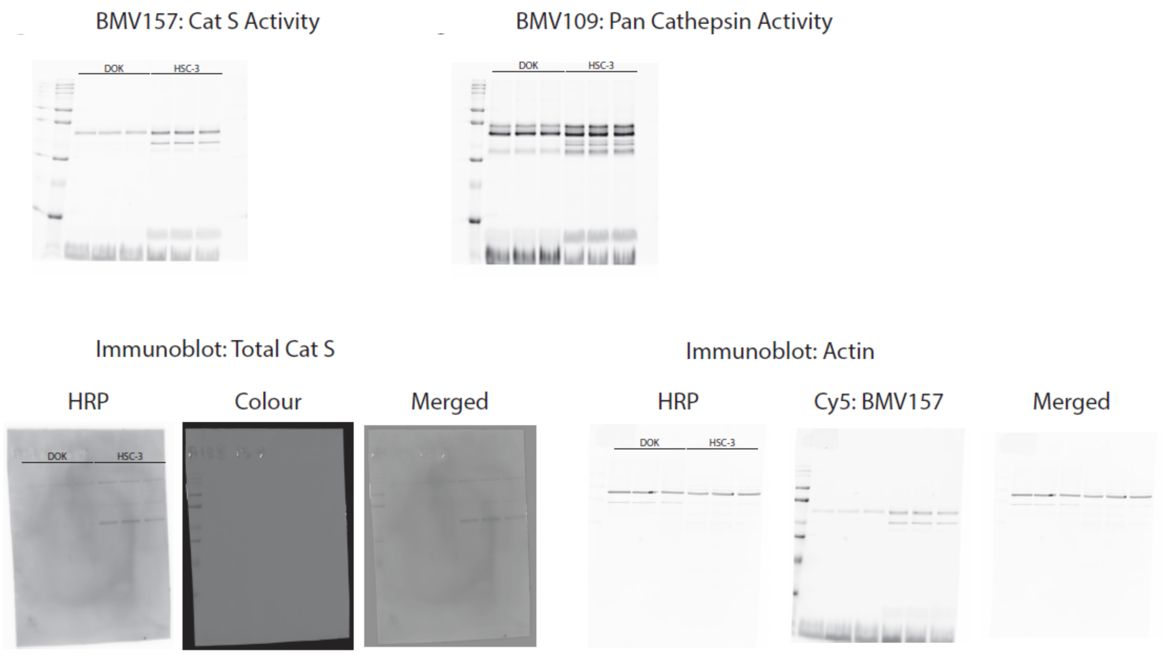

Figure S3. Uncropped western blot figures of Figure 6a.
